# A Giant Sporadic Intra-abdominal Desmoid Tumor in a Male Patient: A Case Report

**DOI:** 10.7759/cureus.26633

**Published:** 2022-07-07

**Authors:** Basma Elhaddad, Dheeraj Gopireddy, Shiguang Liu

**Affiliations:** 1 Department of Pathology and Laboratory Medicine, University of Florida College of Medicine – Jacksonville, Jacksonville, USA; 2 Department of Radiology, University of Florida College of Medicine – Jacksonville, Jacksonville, USA

**Keywords:** case report, intra-abdominal fibromatosis, differential diagnosis, large intra‑abdominal mass, desmoid tumors

## Abstract

Desmoid tumors (DTs) are rare locally aggressive benign soft tissue tumors with an estimated annual incidence of two to four new cases per million people. The giant intra-abdominal mass presents a diagnostic challenge that includes a broad differential diagnosis of gastrointestinal stromal tumor (GIST), fibrosarcoma, retroperitoneal fibrosis, and other malignancies from adjacent organs. We report a case of a 38-year-old male patient with a giant intra‑abdominal mass. Magnetic resonance imaging (MRI) of the abdomen and pelvis indicated mucinous cystic neoplasm or gastrointestinal stromal tumor (GIST), but histopathology confirmed it to be a desmoid tumor. The patient was discharged, and on follow-up five months until now, there is no recurrence. This case highlighted the importance of including DT in the differential diagnosis of very large intra‑abdominal masses.

## Introduction

Desmoid tumors (DTs) are rare locally aggressive benign soft tissue tumors with an estimated annual incidence of two to four new cases per million people in the general population [1]. Giant mesenteric DTs are even rarer. In the literature search, we only found six case reports of giant intra-abdominal DT measuring greater than 20 cm but less than 40 cm in the greatest linear dimension [2-7]. DT can arise from the abdominal wall but rarely arise from abdominal organs or mesentery [1]. We report a case of a giant desmoid tumor arising from the ileocecal mesentery requiring surgical resection due to mass effect.

## Case presentation

**Figure 1:**
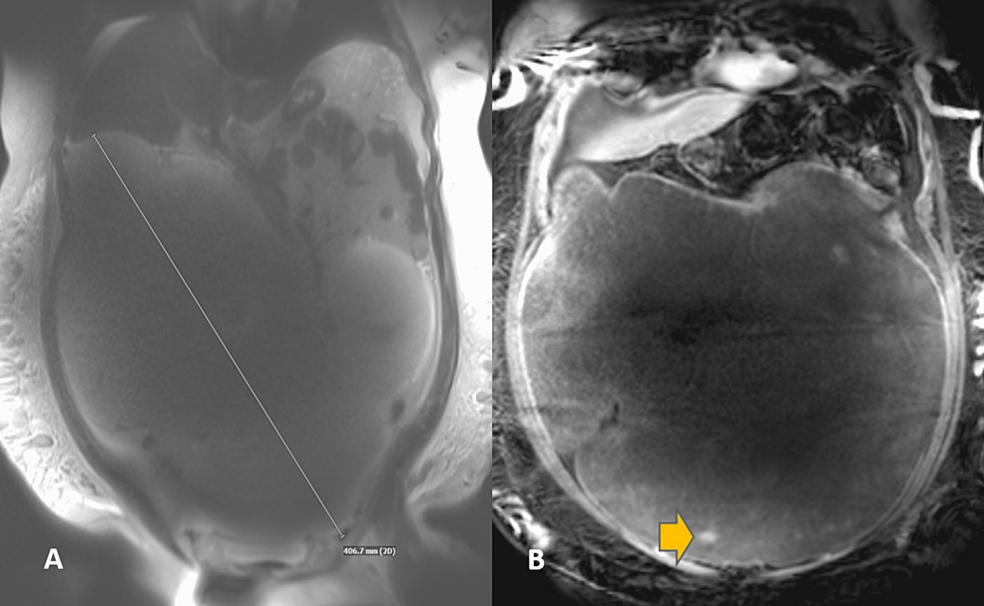
Coronal T2-weighted MR image A: Coronal T2-weighted image showing a giant infra-mesocolic abdominal mass measuring close to 40 cm. B: Coronal post-contrast image showing areas of solid enhancement (arrow) within the mass.

A 38-year-old male patient presented with progressive abdominal pain, swelling, and dyspnea on exertion for two to three months. Physical examination showed a massively distended, firm, but non-tender abdomen. He also had moderate pitting edema of bilateral lower extremities but was otherwise unremarkable. He denied fevers, chills, weight loss, night sweats, changes in bowel habits, or hematochezia. Serum CEA, CA-19-9, and AFP were all negative. He had no family history of colon cancer, familial adenomatous polyposis (FAP), or personal history of abdominal trauma. Computed tomography (CT) scan showed a massive peritoneal mixed density (solid and cystic components) mass encasing the ascending colon. Magnetic resonance imaging (MRI) was suggested to further elucidate the origin of this mass (Figure 1).

The mass was identified to extend from the pelvis to the upper abdomen and measures approximately 19 × 40 × 30 cm (anteroposterior × transverse × craniocaudal). It was predominantly infra-mesocolon and demonstrated areas of small solid enhancement. Based on imaging, the tumor was most likely arising from the right lower quadrant with encasement of the cecum and vermiform appendix. Considering the predominant T2 signal on MRI, the differential diagnosis included mucinous cystic neoplasm versus gastrointestinal stromal tumor (GIST). The patient underwent an elective exploratory laparotomy to remove a giant intra-abdominal mass occupying the whole abdomen and was densely adherent to the ileocecum. Right partial colectomy with ileocolonic anastomosis and ileocolostomy were performed after the mass was removed.

**Figure 2:**
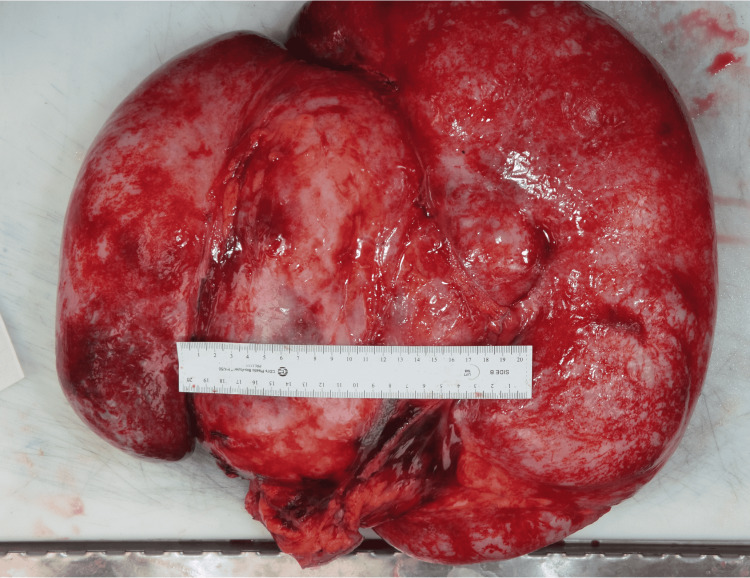
Intra-abdominal mass (gross image) A giant, well-circumscribed, firm mass weighing 12,804 g and measuring 40 × 34 × 15 cm.

**Figure 3:**
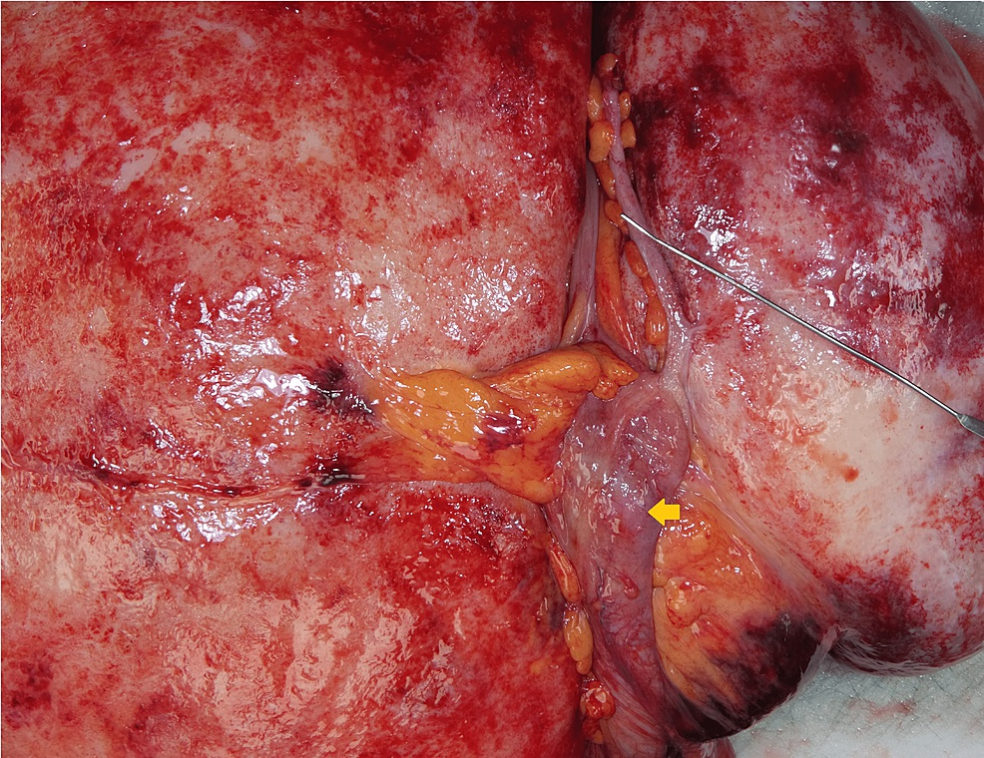
Intra-abdominal mass (gross image) The mass was attached to the ileocecum and appendix. The probe indicated the vermiform appendix, and the arrow indicated the ileocecum.

The macroscopic pathological examination showed a very large, well-circumscribed, firm mass weighing 12,804 g and measuring 40 × 34 × 15 cm. The mass revealed a smooth external surface and was attached to the ileocecum and appendix (Figures 2, 3).

**Figure 4:**
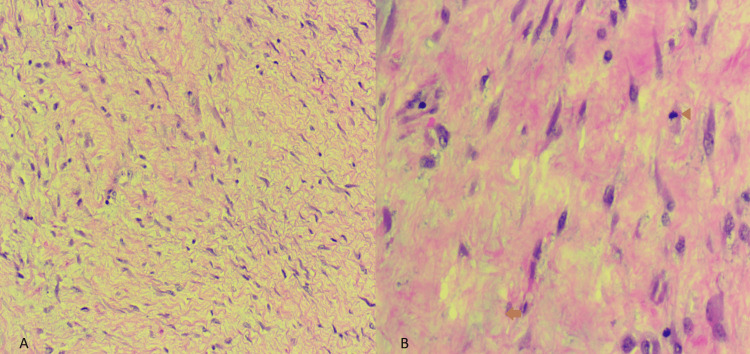
Histology (hematoxylin and eosin stain) (A) The tumor was composed of uniform, bland spindled to stellate cells with low cellularity. (B) The stroma was collagenous with occasional myxoid changes (arrow). No necrosis and hemorrhage were identified. Mitosis is <1/10 HPF (arrowhead).

Histopathology showed low cellularity composing of uniform bland of spindled to stellate tumor cells with small vesicular nuclei. These cells contained several tiny nucleoli and low mitoses (<1/10 HPFs). No tumor necrosis and hemorrhage were identified. Stroma was collagenous with occasional myxoid changes (Figure 4). These findings were consistent with the diagnosis of a benign desmoid tumor. The vermiform appendix, colon, and ileum were unremarkable upon examination.

**Figure 5:**
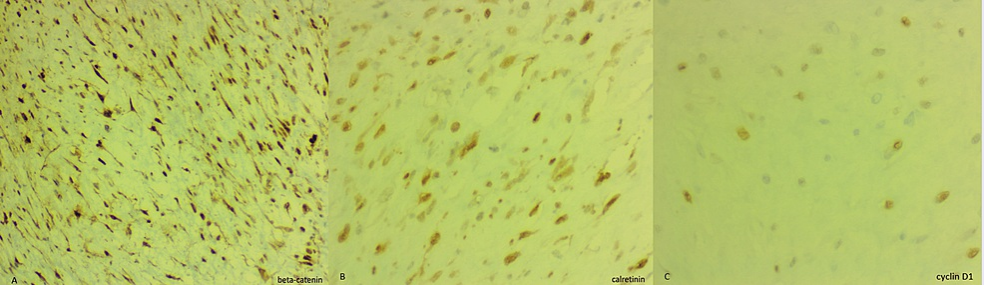
Immunohistochemistry (beta-catenin, calretinin, and cyclin D1) (A) Strong immunostaining for nuclear beta-catenin. (B) Nuclear and cytoplasmic immunostaining for calretinin. (C) Partial nuclear staining for cyclin D1.

**Figure 6:**
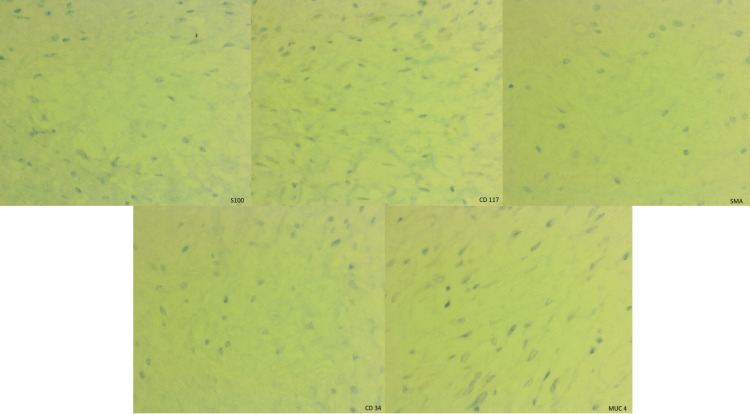
Immunohistochemistry (S100, CD 117, SMA, CD 34, and MUC4) Negative immunostaining for S100, CD 117, SMA, CD 34, and MUC4.

Immunohistochemical stains showed diffuse staining of beta-catenin, calretinin, and partial cyclin D1 (Figure 5) and negative staining of S100, CD 117, smooth muscle actin (SMA), CD 34, and MUC4 (Figure 6).

The patient's postoperative course was complicated by the development of multiple intra-abdominal abscesses that required drain placement by interventional radiology. The patient also was found to have Gram-negative bacteremia (*Bacteroides fragilis*, sensitive to piperacillin and tazobactam) requiring central line placement, intravenous infusion of piperacillin and tazobactam, and total parenteral nutrition (TPN). He was discharged after successful treatment of the abovementioned complications. Postoperative follow-up has been five months until the paper submission with no recurrence.

## Discussion

Desmoid tumors are locally aggressive fibroblastic/myofibroblastic tumors arising in deep soft tissue with no metastatic potential. It is characterized by a high recurrence rate due to infiltration of the surrounding tissue [2,3]. The mainstay of treatment is surgical removal with negative margins. Desmoid tumors are classified as abdominal, extra-abdominal, and intra-abdominal types. The most frequent site of intra-abdominal desmoid tumors is small bowel mesentery. These tumors are more common in females and are thought to be associated with estrogen [4]. It can occur sporadically or can be associated with familial adenomatous polyposis (Gardner syndrome) [8,9]. Hence, regular screening colonoscopy is recommended in addition to regular follow-ups.

Differential diagnoses for solid mesenteric masses are comprehensive, which include mesenteric lymphoma, metastatic disease, carcinoid tumors, desmoid tumors, gastrointestinal stromal tumors (GISTs), liposarcoma [9], low-grade fibromyxoid sarcoma, sclerosing mesenteritis, neurogenic tumors, leiomyoma, and solitary fibrous tumors [10]. The clinical history of diagnosed cancer may help assess the metastatic disease. Immunohistochemical markers play an essential role in identifying and differentiating diagnoses. Primary mesenteric liposarcomas are very rare and may have predominant non-lipogenic components forming solid mesenteric masses. Well-differentiated and dedifferentiated liposarcomas show MDM-2 gene amplification. Sclerosing mesenteritis is distinguished from mesenteric fibromatosis by an abundance of inflammation and fat entrapment with fat necrosis [10]. CD 117 positivity in fibromatosis is often seen in cytoplasm versus membranous and cytoplasmic positivity in GIST. GISTs lack nuclear beta-catenin staining as it is present in fibromatosis. Low-grade fibromyxoid sarcoma is positive for MUC4 and FUS-CREB3L2 translocation. Inflammatory myofibroblastic tumors typically occur in younger age groups [7], and a component of inflammation is present with ALK1 gene rearrangements (in 50% of cases). Leiomyoma is composed of smooth muscle cells and is positive for SMA and desmin. The solitary fibrous tumor is diffusely positive for CD 34 and STAT 6. Neurogenic tumors and neuroendocrine cell tumors including carcinoid tumors are positive for neural markers and neuroendocrine markers, respectively. Our case shows diffuse nuclear staining of beta-catenin, nuclear and cytoplasmic immunostaining of calretinin, and partial nuclear staining of cyclin D1. Immunostaining for S100, CD 117, SMA, CD 34, and MUC4 are negative. Due to the typical morphology and immunostaining pattern, a diagnosis of a desmoid tumor was rendered. No molecular study was performed for this case.

Surgical excision is the primary option for the treatment of intra-abdominal DT. Postoperative radiotherapy, chemotherapy, or a combination of the two has been used in cases of positive margins with variable successes [11]. The recurrence of DT after surgical removal ranges from 19% to 57%, with a median disease-free survival of between 14 and 24 months [12]. Desmoid tumor in Gardner syndrome is associated with higher recurrence rates. Most patients have a significant family history and numerous small and large polyps revealed in the excised colon, which was not consistent with this patient.

## Conclusions

We present a very rare, giant intra-abdominal DT in a male patient, which is the largest one in the reported literature to date. Differential diagnoses for giant intra-abdominal masses are very broad, including DT and inflammatory conditions, and benign and malignant neoplasms. Clinical history, histology features, immunohistochemical tests, and, in some cases, molecular studies are important to solve this challenging diagnosis.
